# Extracellular Matrix Structure and Interaction with Immune Cells in Adult Astrocytic Tumors

**DOI:** 10.1007/s10571-024-01488-z

**Published:** 2024-07-05

**Authors:** Anna Di Vito, Annalidia Donato, Jessica Bria, Francesco Conforti, Domenico La Torre, Natalia Malara, Giuseppe Donato

**Affiliations:** 1 Department of Clinical and Experimental Medicine, University Magna Graecia of Catanzaro, Catanzaro, Italy; 2 Department of Health Sciences, University Magna Graecia of Catanzaro, Catanzaro, Italy; 3 Pathology Unit, “Annunziata” Hospital, Cosenza, Italy; 4 Unit of Neurosurgery, Department of Medical and Surgical Sciences, University Magna Graecia of Catanzaro, Catanzaro, Italy

**Keywords:** Adult astrocytic tumors, Extracellular matrix, Hyaluronic acid, Collagen, Periostin, Tenascin C, Matrix metalloproteases, Macrophages, Lymphocytes

## Abstract

The extracellular matrix (ECM) is a dynamic set of molecules produced by the cellular component of normal and pathological tissues of the embryo and adult. ECM acts as critical regulator in various biological processes such as differentiation, cell proliferation, angiogenesis, and immune control. The most frequent primary brain tumors are gliomas and by far the majority are adult astrocytic tumors (AATs). The prognosis for patients with these neoplasms is poor and the treatments modestly improves survival. In the literature, there is a fair number of studies concerning the composition of the ECM in AATs, while the number of studies relating the composition of the ECM with the immune regulation is smaller. Circulating ECM proteins have emerged as a promising biomarker that reflect the general immune landscape of tumor microenvironment and may represent a useful tool in assessing disease activity. Given the importance it can have for therapeutic and prognostic purposes, the aim of our study is to summarize the biological properties of ECM components and their effects on the tumor microenvironment and to provide an overview of the interactions between major ECM proteins and immune cells in AATs. As the field of immunotherapy in glioma is quickly expanding, we retain that current data together with future studies on ECM organization and functions in glioma will provide important insights into the tuning of immunotherapeutic approaches.

**Graphical Abstract:**

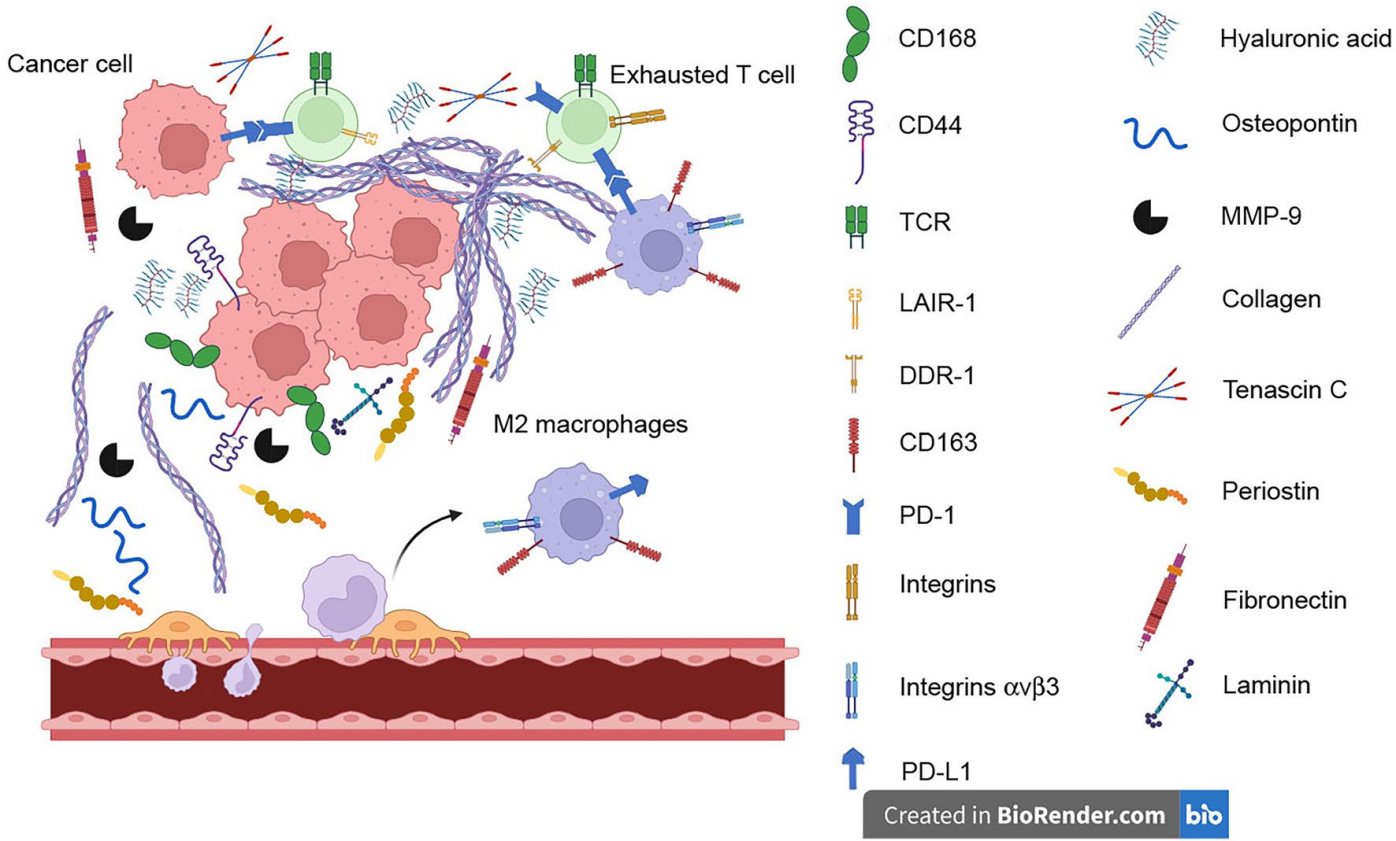

## Introduction

The WHO 2021 classification of Tumors of the Central Nervous System (WHO CNS5) relies on three different approaches: histology, immunohistochemistry, and molecular profiling (Louis et al. 2021). Gliomas, glioneuronal tumors, and neuronal tumors are recognized as the most frequent and most heterogeneous tumors affecting the parenchyma of the CNS. Adult-type diffuse gliomas, and mainly glioblastoma (GBM) IDH-wildtype and astrocytoma IDH-mutant represent the most important neoplasms in the practice of adult neuro-oncology (Table 1) (Śledzińska et al. 2021).

**Table 1:** Gliomas according to the 2021 World Health Organization Classification of Tumors of the Central Nervous System (Śledzińska et al. 2021)

Families	Types
Adult-type diffuse gliomas	Astrocytoma, IDH-mutant Oligodendroglioma, IDH-mutant, and 1p/19q-codeleted Glioblastoma, IDH-wildtype
Pediatric type diffuse low-grade gliomas	Diffuse astrocytoma, MYB- or MYBL1-altered Angiocentric glioma Polymorphous low-grade neuroepithelial tumor of the young Diffuse low-grade glioma, MAPK pathway-altered
Pediatric type diffuse high-grade gliomas	Diffuse mildline glioma, H3 K27-altered Diffuse hemispheric glioma, H3 G34-mutant Diffuse pediatric-type high-grade glioma, H3-wildtype and IDH-wildtype Infant-type hemispheric glioma
Circumscribed astrocytic gliomas	Pilocytic astrocytoma High-grade astrocytoma with piloid features Pleomorphic xanthoastrocytoma Subependymal giant cell astrocytoma Chordoid glioma Astroblastoma, MN1-altered

According to the Central Brain Tumor Registry of the United States (CBTRUS) the most commonly occurring malignant CNS tumor is glioblastoma (14.5% of all tumors) (Ostrom et al. 2020). On the other hand, the data concerning the IDH-mutant astrocytoma are not clear due to the recent change in nomenclature. The WHO CNS5 does not consider immunology of glioma. An immune suppressive microenvironment characterizes many types of brain tumor which for this reason are defined “immunologically cold.” Many data in recent years have highlighted the importance of the “normalization” of tumor microenvironment to restore host immunity, as well as the advantages of immunotherapeutic approaches (Finch et al. 2021; Guadagno et al. 2018; Mignogna et al. 2016; Presta et al. 2018; Vismara et al. 2019).

In general, the most frequent primary brain tumors are gliomas and by far the majority are adult astrocytic tumors (AATs) which are included in the adult-type diffuse glioma family. The prognosis for patients with these neoplasms is poor and the treatments provide only modest results in terms of overall survival and disease-free survival. AATs are among the most challenging tumors to treat due to their well-known resistance to conventional therapies.

Extracellular matrix (ECM), also known as the matrisome, is a dynamic set of molecules produced by the cellular component of normal and pathological tissues of the embryo and adult. ECM acts as critical regulator in various biological processes such as differentiation, cell proliferation, angiogenesis, and immune control. Given the fact that the cancer cells heterogeneity, genomic instability, and the development of drug resistance are the most common challenges in therapies targeting cancer cells, the identification of new targets in tumor microenvironment such as ECM components, could provide a valuable alternative with reduced off-target effects. Indeed, ECM molecules are more stable and much less prone to mutations or altered expression than cell membrane receptors in response to chemotherapy or radiotherapy. Unfortunately, despite many studies have been devoted to the ECM of malignant solid tumors in humans, ECM composition in AATs remains poorly understood.

Recently, evidence has emerged that the ECM conveys specific signals to cells, thereby modulating immune cell functions. ECM-immune cells communication take place via several mechanisms, including direct interaction via cell surface-binding proteins, storage, and release of secreted molecules with immunomodulatory properties, the recruitment of immunity cells by ECM fragments. On the other hand, ECM composition around the tumor undergoes profound changes during tumor progression and specific immune cells seem to contribute to that. Hence, the ECM and the immune system are intertwined, and both contribute to shaping the tumor immune microenvironment.

Innate immune cells and adaptive immune cells involved in oncogenesis and glioma progression are summarized in the Table 2. Despite a clear description of immune cells in glioma has been provided, many studies have highlighted some differences between IDH-mutated and IDH-wildtype tumors. In IDH-mutated glioma, the oncometabolite (R)-2-hydroxyglutarate (R-2-HG) plays both direct and indirect actions. In particular, R-2-HG accumulation results in suppression of the innate immune systems (macrophages, dendritic cells, natural killer cells) and adaptive immune cells, especially T cells while the specific regulation of B cells remains still poor understood (Cai et al. 2024; Stepanenko et al. 2024).

**Table 2:** Innate immune cells and adaptive immune cells involved in glioma development and progression

Immune cells	Main features	Basic references
Tumor-associated macrophages (TAMS)	TAMS derive from circulating macrophages and/or from microglia and are the dominant population of immune cells in the glioblastoma microenvironment. In gliomas around 30–50% of cells are macrophages or activated microglia and up to 70% of the total cell populations of GBM tissues may be composed of macrophage/microglia. Macrophages classically activated by IFNγ and PAMPs or DAMPs produce high levels of pro-inflammatory cytokines like IL-12 and are called M1 macrophages. They promote antitumor immune responses. However, in tumor and transplant sites is present a second type of macrophages called M2 macrophages, they downregulate expression of MHC class II and produce IL-4, IL-10, and IL-13. M2 macrophages balance the effects of M1, support tumor angiogenesis, metastasis, and cancer cells invasiveness The polarization state of TAMS, M1 or M2, is a continuum that reflects the ability of these cells to act in an anti-inflammatory and pro-tumor sense (M2) or in a pro-inflammatory and anti-tumor sense (M1) TAMS in AATs are M2 polarized producing a “cold” environment	Guadagno et al. (2018) Stepanenko et al. (2024) Sica, Mantovani (2012) Martinez et al. (2014)
Cytotoxic T cells and TILs	The main protective mechanism of adaptive immunity against tumors is the killing of tumor cells by CD8+ cytotoxic T lymphocytes (CTL) The mononuclear cells observed in the inflammatory infiltrate of solid tumors, called Tumor-Infiltrating Lymphocytes (TILs), include CTLs capable of killing neoplastic cells, CD4+T cells, B cells, and other lymphoid lineage cells like NK and NKT cells Specific CD8+T responses to tumor antigens require cross-participation by dendritic cells Programmed cell death protein-1 (PD-1) and anti-cytotoxic T lymphocyte–associated protein 4 (CTLA-4) are expressed in the mechanism of immune tolerance on such cells. Tumors are able to activate mechanisms that inhibit immune responses also through this type of receptors The density of tumor-infiltrating CD4+, CD8+, and Tregs increases with glioma grade. TILs are not very numerous and seem more represented at the level of the vascular niche than among tumor cells, which is because GBM essentially remains a “cold” tumor that benefits little from therapies such as those relating to inhibitory check points. However, the number of CD8+ cells appears to be positively correlated with the survival of patients with GBM The ratio between immunosuppressive immune cell subsets and cytotoxic lymphocytes plays a prognostic role. Increased CD8+/CD4+, CD4+/CD8+, CD3+/Treg, or CD8+/Treg ratios can be correlated with survival in patients with gliomas NK cells are cytotoxic lymphocytes, capable of direct killing without prior immunization. TAMs can inhibit NK cell activation against tumor cells in a contact-dependent manner via TGF-β Purified NK cells, employed in GBM treatment, may exert a preferential killing of GBM stem-like cells	Vismara et al. (2019) Han et al. (2014) Mauldin et al. (2021) Presta et al. (2018)
Antigen-presenting cells (Dendritic Cells)	Dendritic Cells (DCs) are a heterogenous population composed by several subsets They play a role in starting and regulating the innate and adaptive immuneresponse The role of the dendritic cell compartment in patients with glioblastoma is poorly characterized and evidently complex The GBM microenvironment contains immunosuppressive factors, such as TGF-β and IL-10, which inhibit the maturation and function of DCs However, GBM cells release chemotactic factors, like CCL2 and CXCL12, which can attract specific DC subsets to the neoplasm	Gardam et al. (2023) Hu et al. (2023)
Neutrophils	Neutrophils in cancer can exert pro-tumor or anti-tumor activities and exhibit various polarization phenotypes. Tumor associated neutrophils (TANs) fall in two subsets: N1show an antitumor phenotype, producing reactive oxygen intermediates and reactive nitrogen intermediates which can kill tumor cells, in the absence of TGFβ the N1 neutrophils produce N2O2 and TNF and stimulate CTL cells; N2 produce angiogenetic factors and dissolve the ECM to favor tumor cell migration The level of neutrophil infiltration is significantly correlated with glioma grade. Increased neutrophil infiltration is associated with shorter overall survival in patients with glioblastoma	Shaul Fridlender (2019) Lin et al. (2021) Han et al. (2015)
Myeloid-Derived Suppressor Cells (MDSCs)	MDSCs are pathologically activated neutrophils and monocytes. In fact, there are two major groups of MDSCs in humans and mice, namely granulocytic/polymorphonuclear MDSCs (PMN-MDSCs) and monocytic MDSCs (M-MDSCs), classified according to their origin from the granulocytic or monocytic myeloid cell lineages, respectively. MDSCs are able to inhibit immune responses, including those mediated by T cells, B cells, and natural killer (NK) cells. M-MDSCs and PMN-MDSCs can suppress the immune responses, by the upregulation of signal transducer and activator of transcription 3 (STAT3) expression, induction of Endoplasmic Reticulum stress, expression of arginase 1 and of S100A8/A9 MDSCs induce immunosuppression and promote tumor development in gliomas	Veglia et al. (2021) Mi et al. (2020)
Mast Cells (MCs)	Many studies have revealed that MCs have a pro-tumorigenic or anti-tumorigenic role depending on the type of cancer MCs infiltrate gliomas in response to variety of signals in a glioma grade-dependent manner. ‘Tumor educated’ MC derived IL-6 is involved in the observed MC induced biological effect on glioma cells	Donato et al. (2014) Derakhshani et al. (2019) Mignogna et al. (2017) Attarha et al. (2017)

Based on these considerations, the aim of our study is to review the current knowledge regarding the regulatory circuits that closely link the ECM and the immune system in AATs. The effect of major ECM proteins such as hyaluronic acid (HA), collagen, tenascin C (TNC), periostin, fibronectin, and laminin on immune landscape in glioma is presented. We also evaluate the prognostic value of ECM proteins, and their potential role as therapeutic targets in this field.

## ECM Molecules in AATs

The ECM is a dynamic meshwork consisting of collagens, non-collagenous glycoproteins, and proteoglycans. Resident and immigrant cells in normal and pathological tissues are embedded in the ECM. Every tissue has an ECM with unique physical properties and organization. Both the concentrations of ECM constituents and the role of ECM as reservoir of signaling molecules play a key role in orchestrating cell behavior and morphogenesis. The ECM undergoes remodeling consisting of the synthesis, degradation, and reassembly of its components mediated by matrix-degrading enzymes including matrix metalloproteinases (MMPs). Additionally, both production and activity of MMPs are regulated by extracellular matrix metalloproteinase inducer (EMMPRIN) and tissue inhibitors of MMPs (TIMPs) (Wang et al. 2018). In pathological states such as inflammation and cancer, the ECM is profoundly modified from the norm with alterations that may counteract or contribute to disease progression. The bidirectional crosstalk between ECM and a plethora of cells including cancer cells, immune cells, and endothelial cells, determines the rearrangements observed during cancer progression and involves both physical and humoral interactions.

In this section we will discuss the main ECM alterations observed in AATs and their influence on the immune response to these tumors.

### Hyaluronic Acid

Altered expression of HA and its receptors such as CD44 and CD168 was found to be linked to the genesis and progression of cancer (Donato et al. 1985, 2009; Marotta et al. 1985). Gliomas contain abnormal accumulation of HA and showed overexpression of its receptors, which may facilitate tumor growth by regulating cell migration or by influencing intracellular signaling pathways (Delpech et al. 1993). HA favorites tumor invasion by regulating the release of MMPs (Park et al. 2008). Upon binding to its receptors, HA activates Ras/FAK/ERK 1,2 pathways, induces NFκB nuclear translocation and MMP-9 release. In the nucleus, NFκB activates the promoter of PTK2, the gene encoding FAK, establishing a positive feedback loop. The crucial step in this pathway is the activation of FAK, whose role in tumor invasion, growth, and metastasis is widely accepted (Zeng et al. 2023). As pointed out by (Park et al. 2008), in such a setting PTEN can modulate the expression of MMP-9 by the dephosphorylation of FAK. The high frequency of PTEN loss or mutation in glioma further support the activation of RaS/FAK/ERK 1,2 pathway, cell cycle progression, and metastasis. Therefore, HA overexpression together with PTEN mutation support cancer progression.

HA induces the motility of glioma cells by the induction of osteopontin through the PI3K/AKT/mTOR pathway (Park et al. 2008). Indeed, HA and osteopontin overexpression often correlate and osteopontin can interact with CD44. Also in this case, PTEN mutation further increases glioma malignancy strengthening the action of osteopontin.

Studies on human surgical specimens and cell lines demonstrated that HA metabolism supports glioma growth by regulating autophagy. Beside HA and CD44 upregulation, human glioma tissues display high level of hyaluronic acid synthase 3 (HAS3), which negatively correlated with the prognosis glioma patients. Silencing HAS3 expression or blocking CD44 slowing down glioma cell proliferation, through the inhibition of autophagy and cell cycle arrest in G1 phase (Yan et al. 2021).

The high levels of HA and its receptors in glioma contribute to the immune escape and resistance to immunotherapy. Indeed, GBM cells can “re-educate” tumor-associated macrophages (TAMs) to become cancer-permissive by secreting factors such as HA (Liu et al. 2019). Secreted HA induces the apoptosis of dendritic cells and prevents the production of specific T cells. Moreover, HA-enriched stroma supports the development of PD-L1-expressing macrophages which establish an immunosuppressive environment (Dominguez-Gutierrez et al. 2022).

The therapeutic properties of HA are exploited for the development of a new oncolytic virus in glioma treatment. Oncolytic viruses were firstly developed to target and kill cancer cells but also to create an antitumor microenvironment. Among oncolytic adenoviruses, ICOVIR17 is characterized by an insertion of E2F binding sites in the E1A promoter and the SPAM1 gene encoding PH20 hyaluronidase. Treatment of orthotopic GBM xenografts with ICOVIR17 mediates the degradation of HA in GBM ECM, resulting in increased virus spread within GBM and higher anti-tumor efficacy compared with its parental virus ICOVIR15 without the transgene. Moreover, degradation of HA enhances oncolytic adenovirus immunotherapy of GBM by overcoming the immunosuppressive functions of ECM. Indeed, treatment of murine orthotopic GBM with ICOVIR17 increased tumor-infiltrating CD8+T cells and macrophages, and upregulated PD-L1 on GBM cells and macrophages (Kiyokawa et al. 2019, 2021).

In a mice model of GBM, encapsulated human adipose-derived mesenchymal stem cells loaded with ICOVIR17 in biocompatible synthetic ECM resulted in a significant decrease in tumor regrowth and increased survival compared with direct injection of ICOVIR17 (Martinez-Quintanilla et al. 2015). Notably, there is no clear data on therapeutic potential of MSCs for brain cancer. Although in the research of Martinez-Quintanilla et al. (2015) MSC-based therapy appeared to suppress glioma growth, MSCs from different sources could display different behavior according to their high heterogeneity in clonogenicity and multilineage differentiation potential (Do et al. 2021; Di Vito et al. 2019).

### Collagen

Collagen is the most abundant protein in mammals. Many types of cells, such as fibroblasts, osteoblasts, chondroblasts, epithelial cells, and astrocytes can produce it. The collagen superfamily comprises 28 members consisting of triple helix polypeptide chains, named α chains, numbered by roman numerals. The diversity of the collagen family is due to several α chains and several molecular isoforms. The three α chains can be either identical to form homotrimers (e.g., collagen II) or different to form heterotrimers (e.g., collagen IX) (Di Vito et al. 2021; Hirano et al. 2004).

Collagen is poorly expressed in normal brain; however, it is overexpressed in human GBM and in glioma cell lines. Studies on tissue microarrays of human GBM and Xenografts of Glioblastoma Stem-Like Cells (GSC) in mice also examine collagen’s correlation with patient survival, establishing that patients with more organized GBM collagen survive longer than patients with less organized GBM collagen (Pointer et al. 2017). Recent observations in vitro have established that collagen density is an important regulator of the immunosuppressive phenotype of TAMs. This mechanism could be critical for the ability of cancers enriched in M2 polarized macrophages, such as GBM, to evade immune destruction and could constitute a limiting factor for the efficacy of immunotherapy (Larsen et al. 2020). Moreover, GBM is recognized as a "cold" tumor, i.e., a tumor accompanied by a scarce infiltrate composed of lymphocytes (TILs) (Guadagno et al. 2018). In cancers, T cell number and activity are modulated via a direct binding of collagen to specific T cells receptors, including leukocyte-associated Ig-like receptor-1 (LAIR-1), discoidin domain receptor 1 (DDR1), and several integrins. Furthermore, the induction of immune suppressive activity in other immune cells such as macrophages also contribute to collagen-mediated TIL decrease (Rømer et al. 2021; Scali et al. 2016).

Interestingly, the deposition of both collagen and hyaluronic acid increases the stiffness of ECM and the transmission of physical change signals to the intracellular cytoskeleton. These interactions were responsible for the changes observed in cell metabolism and behavior, including cancer cell proliferation, migration, and genomic instability. A stiffened ECM favor fibrosis development and the formation of a physical barrier to both T cell penetration and drug infiltration, with consequent immunosuppression and drugs resistance (Ge et al. 2021; Flies et al. 2023). Therefore, both abnormal collagen accumulation and poor collagen organization are responsible for the immunosuppressive environment reported in glioma.

Among the different collagen subtypes expressed in glioma, collagen VI was reported to be overexpressed in GBM and high-grade glioma when compared to lower-grade astrocytoma and normal glia (Fujita et al. 2008). Collagen VI is composed of three different subunits, α1(VI), α2(VI), and α3(VI), whose overexpression in GBM relapses has been related to increased DNA repair and cell survival in response to both radio- and chemotherapy (Cescon et al. 2023). The aberrant activation of pro-survival signaling pathways including DNA replication and repair genes by collagen VI favor the development of pro-malignant cell behavior in GBM and negatively impact on GBM patients’ prognosis (Cescon et al. 2023). In particular, collagen VI deposition has been correlated with the enhanced invasion associated with resistance to bevacizumab (Cha et al. 2023). Hence, this relationship suggests collagen VI as therapeutic targets to reduce invasion associated with bevacizumab resistance and thus improve clinical outcomes.

Recently, specific collagen genes have been associated to immune infiltration and cancer progression in glioma (Yin et al. 2021). Higher expression of COL1A1, COL1A2, COL3A1, COL4A1, COL4A2, and COL5A2 was negatively correlated with the prognosis of glioma patients (Yin et al. 2021). Furthermore, collagen genes expression was closely associated with immune cell infiltration in glioma. In detail, COL1A1, COL1A2, COL3A1, COL4A1, and COL4A2 positively correlated with the infiltration of B cells, CD8+T cells, CD4+T cells, macrophages, neutrophils, and dendritic cells in low-grade glioma. In this case, COL5A2 display positive correlation with all immune cells except for CD4+T cells. The six collagen genes were positively correlated with the infiltration of dendritic cells in GBM (Yin et al. 2021). In addition, the expression of the genes showed positive correlation with immunosuppressive properties in glioma microenvironment.

The role played by COL3A1, also known as collagen αlpha-1(III), has been previously investigated by Gao et al (2018). They reported COL3A1 upregulation in glioma cells and showed that COL3AI silencing in vitro was sufficient to inhibit glioma cell proliferation and migration (Gao et al. 2018). Interestingly, miR128-3p was able to modulate the expression of COL3AI in glioma, and the expression of miR128-3p and COL3A1 are inversely correlated. Accordingly, the knockdown of COL3A1 by using small interfering RNA (siRNA) in SHG44 and A172 cells suppressed migration, invasion, and epithelial-mesenchymal transition (EMT) process in glioma cells (Yin et al. 2021).

Collagen alpha-2(I) chain (COL1A2) is a chain of type I collagen whose triple helix comprises two alpha-1 chains and one alpha-2 chain. Wang et al. (2022a, b) reported significant higher expression of COL1A2 in the blood of patients with glioblastoma (GBM) compared with healthy controls. In surgical specimens and human cell lines, COL1A2 inhibition attenuated GBM proliferation by promoting cell cycle arrest, suggesting a key role in GBM progression (Wang et al. 2022a, b).

Recently, plasma level of collagen IV has also been indicated as prognostic marker of glioma malignancy (Oldak et al. 2022).

Altogether, this evidence strongly underlies the need for the identification and validation of new strategies targeting either specific collagen proteins or the collagen meshwork. In the first case, the application of specific miRNA or siRNA that synergize with chemotherapeutics or radiotherapy could represent a valuable approach to improve tumor therapy safety and efficacy. The strategies directed to the ECM stiffness in glioma include drugs targeting TGF-β, recognized as key factor in collagen synthesis, collagenases to directly degrade collagen, and inhibition of collagen cross-linking (reviewed in Huang et al. 2021).

Interestingly, ECM collagen has been indicated as major player in immunotherapy resistance. In an all-encompassing vision of collagen action, it was observed that the cancer phenotype resulting from abnormal collagen production, which interferes with immunity activation acting as a barrier, overlap with phenotype resulting from ECM receptors signaling (i.e., LAIR-1 and Integrins) (Flies et al. 2023). According to this idea, some authors showed a positive association between the accumulation of specific collagen-derived peptides (such as PRO-C3) and a poor prognosis in patients treated with immune checkpoint inhibitors, suggesting that ECM-immune cell receptors could play an important role. Therefore, the above-mentioned interactions between collagen or its derivatives and a plethora of T cells receptors, not only modulate T cells activation but also counteract immunotherapy (He et al. 2021; Flies et al. 2023).

Based on these considerations, therapeutics that target the ECM are strongly needed to strengthen immunotherapy. Recently, three potential strategies to overcome tumor ECM/collagen-mediated immune suppression have been suggested (Flies et al. 2023): (1) development of therapies targeting ECM immune cell receptors; (2) development of combination strategies with therapeutics directed to ECM stiffness and immunotherapy; (3) development of synthetic products obtained by combining therapeutic substances with drugs targeting the ECM components.

### Tenascin C

TNC consists of six identical monomers that are disulfide-linked into a hexamer at their N-termini. Each subunit is approximately 150–250 kDa in humans and is organized in four different parts: an N-terminal cysteine-rich domain involved in oligomerization, 14.5 epidermal growth factor (EGF)-like repeats, eight constitutively expressed fibronectin type III (FNIII) domains, and a single globular fibrinogen-like domain at the C-terminus (Abedsaeidi et al. 2023). Several molecular isoforms can be produced through alternative splicing. Post-translation modification can give rise to proteins with different functions. Notably, the small fragment obtained by proteolytic processing displays functions that can be very different from those of the full-length protein (Fu et al. 2022). In the embryo, TNC is widely expressed in the sites of morphogenesis around motile cells, and at different degrees in connective tissues, such as bone and cartilage, and in the central nervous system. Its expression peaks during perinatal stage and then decreases progressively. In the adult human organism, TNC is expressed only in connective tissues that bear tensile strength, below some epithelia and in stem cell niches, such as bone marrow, crypts of the intestine, bulge of hair follicles and bone marrow. In addition, TNC is also expressed in adult brain in both stem cells niches (astrocytes in the subventricular zone and granule cells in the hippocampus) and cerebellum (Fu et al. 2022). TNC belongs to Tenascin family which includes Tenascin-W, Tenascin-X, and Tenascin-R. These and other nonstructural proteins such as periostin ad SPARC are indicated as matricellular proteins which modulate cellular behavior by interacting with other structural and nonstructural ECM components: fibronectin, collagen, periostin, and fibrillin-2 as well as PGs of the lectican family and perlecan. In adult, TNC is re-expressed ectopically in a variety of pathologic situations, such as neuroinflammation, neurodegeneration, ischemia, and solid tumors including brain cancer (Di Vito et al. 2021; Donato et al. 1997, 2009; Fu et al. 2022). The highest level of TNC reported in glioma correlates with tumor grade. Unlike most solid tumors, where fibroblasts represent the main source of TNC and cancer cells are unable to produce it, glioma cancer cells produce high amount of TNC which in turn induces a more malignant glioma phenotype, thus establishing positive feedback (Yalcin et al. 2020).

TNC plays a role in the immunomodulation. Serum TNC might be an indicator of the immunosuppressive microenvironment of low-grade gliomas and a predictive biomarker of the efficacy of immunotherapy (Zhang et al. 2022a, b). It is accepted that TNC regulates both innate and adaptive immunity. Many studies showed that TNC exerts immunosuppressive effects on T cells, reducing T cell activation and migration through blood–brain-barrier in glioma. These effects toward local and distant T cells seem to be mediated by the release of TNC in cancer cell-derived exosomes (Huang et al. 2010; Mirzaei et al. 2018). The role of TNC in the modulation of innate immunity in astrocytic tumors is poor clear. Interestingly, TNC activates both microglia and macrophages and induces M1 polarization. Despite this observation suggests an anti-tumor function for TNC, in vivo and in vitro studies confirmed a pro-tumorigenic action. One possible explanation is that TNC exerts antitumor action during the early phase of cancer development, as previously reported for microglia activity. During cancer progression, the establishment of immunosuppressive microenvironment might derive from both a proper switch from M1-polarized macrophages to M2-polarized macrophages, and TNC overexpression. In this scenario, recent studies demonstrated a role for the modulation of CD47 expression, a “don’t eat me” receptor expressed on glioma cells, in the recruitments of M2-like TAMs and in the upregulation of TNC expression via Notch pathway-mediated mechanism. Although these events facilitate phagocytosis, the increased expression of TNC induce the release of proinflammatory cytokines via a toll-like receptor 4 (TLR4) and STAT3-dependent mechanism in human macrophages (Ma et al. 2019). The increased TNC expression in turn accounts for increased infiltration of immunosuppressive cells such as M2-polarized macrophages and regulatory T cells (Tregs) (Zhang et al. 2022a, b). The potentiality of TNC as therapeutic target has been previously evaluated. Many strategies aimed to silence or decrease TNC expression have been developed and detailed in (Fu et al. 2022).

### Periostin

Periostin is a multi-domain secretory protein of around 90 kDa composed of an amino-terminal EMI domain, a tandem repeat of four FAS 1 domains, and a carboxyl-terminal domain (CTD) (Kii et al. 2017). In human, 10 splice variants have been described with different spatiotemporal expression. Given to its multidomain structure, periostin functions as a scaffold for the assembly of several ECM proteins such as collagens, TNC, laminin, and fibronectin. For example, periostin induces the integration of TNC in ECM as well as collagen cross-linking by interacting with BMP1/2 (Huizer et al. 2020). Interestingly, this function can be modulated during development. Periostin, firstly recognized as a protein specifically expressed in periosteal tissues, is expressed in a wide variety of normal adult and fetal tissues, under stress conditions and in cancer (Di Vito et al. 2015; Kii et al. 2017). Periostin modulates cancer cells proliferation, migration, and EMT, by its direct binding to integrins expressed on cancer cell surface. An overactivation of Akt/PKB, Wnt, and FAK-mediated pathways seems to be required for proper functioning. Periostin expression levels directly correlate with tumor grade and recurrence in all grades of adult human glioma (Mikheev et al. 2015). Similarly, an inverse correlation is reported between periostin expression and overall survival in glioma. Beside the well-recognized role in glioma cancer cells behavior, periostin also plays a role in angiogenesis and immune evasion (Huizer et al. 2020).

In GBM, periostin expression has been reported in both glioma stem cells and pericytes localized in the perivascular niches. Upon secreted, periostin accounts for microenvironment remodeling via interaction with ECM components such as osteopontin. This aspect appears very interesting given the well-known role of osteopontin in fibrillar collagen network organization and then in ECM stiffness (Leung et al. 2013). Furthermore, a strong correlation between osteopontin expression and the histological grade of astrocytomas has been reported (Toy et al. 2009). Periostin stimulates monocytes recruitment and drives M2-polarization which in turn counteract T lymphocytes antitumor action (Shi et al. 2015). Accordingly, knockout of periostin in GSCs significantly reduces TAMs infiltration, inhibits tumor growth, and increases survival in mice with GSCs-derived xenografts (Xu et al. 2022). The therapeutic potential of periostin has been introduced. The development of anti-periostin neutralizing antibodies has produced good results in mouse model of breast and ovarian cancer but no data are available for glioma therapy. Recently, miR-599 has been described as inhibitor of periostin expression and then as potential target to inhibit glioma cell motility and invasion (Zhang et al. 2017).

### Fibronectin

Fibronectin was first characterized in the late 1940s as a high molecular weight plasma protein produced by the liver. Fibronectin is a dimer composed of two ~ 250 kDa subunits covalently linked at their C-termini by a pair of disulfide bonds. Each monomer consists of three types of repeating units: type I, type II, and type III. Type I and II modules display internal disulfide bonds, while type III repeats of fibronectin lack disulfide bonds, conferring elasticity to the fibrils and the ability to modulate fibril rigidity. This ubiquitous protein connects various structural proteins and is increasingly recognized as an important regulator of ECM stiffness. Fibronectin also promotes cell adhesion, migration, growth, and differentiation by interacting with various cell membrane receptors (Dalton, Lemmon, 2021). Classic immunofluorescent studies revealed fibronectin arrangement, highlighting an extensive fibrillar network on the surface of fibroblasts. A similar organization is observed around astrocytes and Schwann cells in CNS (Pankov et al. 2002; Pearlstein et al. 1980).

In GBM, fibronectin expression inversely correlates with patients’ survival (Wu et al. 2022). Fibronectins are among the most upregulated genes in brain glioma compared to healthy tissue (Oldak et al. 2022). The mis regulation of fibronectin interactions with other ECM components, such as collagens and tenascin C, promotes scarring, tumorigenesis, fibrosis, and developmental defects. As a matter of the fact, antibody targeting the collagen-binding site in fibronectin could suppress the fibrillogenesis of collagen. In addition to its interaction with various structural proteins, fibronectin regulates cancer cells behavior via direct binding to integrins and syndecans on the cell membrane. Fibronectin can bind 11 different integrins, among which at least 8 (α5β1, αIIbβ3, α8β1, and all the αv-class integrins) bind an Arginine-Glycine-Aspartate (RGD). In addition, α5β1, αIIbβ3 also bind to a synergy site, whose function is to re-enforce cell adhesion (Benito-Jardón et al. 2017). On the other hand, syndecans-fibronectin interaction plays a key role in the formation of focal adhesions and cytoskeleton rearrangement, thus reinforcing integrin signaling and fibronectin fibrils assembly. Among the different integrins involved in fibronectin fibrils assembly, α5β1 are the most studied. After integrins binding to fibronectin, α5β1-αv-class integrins crosstalk allows cell adaptation to fibrils tension and the activation of focal adhesion kinase (FAK). In addition, α5β1 integrins crosstalks with Syndecan-4 and tissue transglutaminase 2 (TG2) to modulate cell migration and proliferation (Ahn et al. 2023; Guerrero-Barberà et al. 2024).

Data regarding the regulation of the immune response in gliomas by fibronectin are scarce. A few studies indicated fibronectin type III domain containing 3B (FNDC3B, also named FAD104) as potential prognostic marker of GBM. FNDC3B is an endoplasmic reticulum transmembrane protein with a single transmembrane domain at the C terminus and nine repeated fibronectin type III domains, with a well-known role in bone formation and adipocyte differentiation. The overexpression of FNDC3B reported in several types of human cancers, including gliomas, has been associated to a dysregulation of EMT and increased cells proliferation. FNDC3B strongly correlates with immune infiltration in gliomas, modulating above all cytotoxic T cells and antitumor associated immune cells. Moreover, FNDC3B has been suggested as a regulatory factor of various immune checkpoints in glioma (Wang et al. 2022a, b).

Therapies targeting fibronectin have been proposed for a long time. Most of these therapies have been directed to the extra domain A (EDA) and extra domain B (EDB) of fibronectin, which are expressed during vasculogenesis in the embryo and re-expressed during tumor angiogenesis and matrix remodeling in adults. The strong correlation between EDB expression and angiogenesis suggested the possibility to develop vaccines able to direct an immune response toward cancer vasculature (Femel et al. 2014). Moreover, targeting EDA and EBD was suggested for the imaging of tumor lesions in both animal and human cancers by using noninvasive (SPECT or PET) methods (Huang et al. 2021). Numerous antibody–cytokine fusion proteins targeting EDB have been developed. The first developed BC-1 and L19 fused with IL12 and IL2, respectively, showed inhibitory effects on colon, skin, and prostate cancer xenografts. Furthermore, therapeutic effects were reported in melanoma or renal cancer patients treated with either BC-1 or L19 drugs (Eigentler et al. 2011). Most of clinical trials that evaluate L19 bound to different therapeutic agent has been reviewed by Lieverse et al (2020).

More recently, a high-affinity peptide targeting EDB named APT_EDB_ (aptide-conjugated liposome targeting EDB) has been developed for glioma therapy. ATP_EDB_ resulted effective in the intratumoral delivery of drugs in mice models (Saw et al. 2013, 2018).

Interestingly, EDA and EDB have been indicated as potential targets for solid tumors therapy with CAR-T cells (Martín-Otal et al. 2022; Zhang et al. 2022a, b).

Recently, a hydrogel consisting of high-molecular-weight hyaluronic acid (HMW-HA) and an inhibitory fibronectin peptide Arg-Gly-Asp-Ser (RGDS) was developed as therapeutic strategy in GBM treatment. The developed matrix allows the attachment of surrounding cancer cells but inhibits fibronectin activation. In vitro analysis showed that the functionalized hydrogels were cytotoxic, and this effect was further enhanced after the addition of liposomes encapsulating doxorubicin (DOX). Furthermore, the functionalized hydrogels efficiently damaged GBM cells without affecting astrocyte viability, suggesting fibronectin inhibition as a promising therapeutic strategy (Castro-Ribeiro et al. 2024).

### Laminins

Laminins are a family of cross-shaped protein with molecular weights of 500–800 kDa, consisting of three chains: α chain, β chain, and γ chain. Sixteen trimeric isoforms are described in the basement membranes of human tissues. Laminins can associate with each other to form a mesh-like polymer. Laminins actively modulate cell behavior by regulating differentiation, migration, and phenotype stability. Moreover, they also inhibit apoptosis by signaling through cell membrane receptors such as dystroglycan and integrins (Halper et al. 2014).

During glioma progression, laminin-8 (α4 β1 γ 1) is highly upregulated and replaces laminin-9 (α4 β2 γ1). Gliomas with higher expression of laminin 8 exhibit accelerated cellular spread and tumor recurrence (Ljubimova et al. 2006). Moreover, GBM tumors express α2, α3, α4, and α5 laminins chains; α3 and α5 laminins enhance glioma cell migration by binding to integrins (Kawataki et al. 2007). Recently, high or very high levels of laminin-5 have been reported in blood plasma of patients diagnosed with glioma in all grades (Oldak et al. 2022). Laminin-5 α2 chain overexpression is associated with invasiveness of glioma by promoting the migration of cancer cells. Despite the plasma level is not correlated with tumor size, laminin-5 is recognized as prognostic marker of brain glioma. Furthermore, laminin-5 expression correlates with the expression of fibronectin and collagen IV, and all together are indicated as effective markers of brain glioma (Oldak et al. 2022).

In vitro studies on astrocytic tumor cell lines (C6 Rat Glioma) suggest that laminins produced by the tumor play a key role in the activation of microglia in a proinflammatory sense in gliomas via the NF-kB DNA binding activity (Kim et al. 2008).

### Metalloproteinases

The ECM can be degraded by MMPs, a family of 23 zinc-dependent proteases secreted as inactive enzymes and proteolytically activated in the ECM. The activity of MMPs in the extracellular space can be specifically balanced by EMMPRIN and TIMPs (Wang et al. 2018).

MMP-2 and MMP-9 are gelatinases that can degrade many ECM molecules including types IV, V, and XI collagen, laminin, and aggrecan core protein. Like collagenases, MMP-2, but not MMP-9, can digest type I, II, and III collagens. Expression of MMP-2 and MMP-9 positively correlates with glioma grade. Immunohistochemical analysis showed cytoplasmic localization in glioma cells, ECM, basement membrane, and endothelial cells (Virtuoso et al. 2022; Wang et al. 2003). Among the several MMPs involved in glioma progression, MMP-9 has been extensively studied. MMP-9 is regulated by EGFR and modulate many signaling pathways, including PI3K/Akt, STAT3/5, NF-kB, ERK, and SHH (Quesnel et al. 2022). An increased expression of MMP-1, MMP-11, and MMP-19 was also observed during the development and progression of human AATs (Stojic et al. 2008).

Although MMPs were initially discovered and described in macrophages and neutrophils which are essential components in AATs, scientific literature largely refers to tumor cells as a major source of proteases which can act on the tumor ECM generating a space for cell mobility (Amălinei et al. 2007; Chou et al. 2016). Similar mechanism has been proposed to explain the contribution of glutamate signaling in glioma progression. Indeed, in glioma the high extracellular concentration of glutamate lead to neurons death, according to the well-known mechanism of “glutamate excitotoxicity” (Di Vito et al. 2014; Neves et al. 2023), generating a space that might be permissive to astrocytoma cells invasiveness.

Both macrophages- and neutrophils-derived MMPs and glioma cells-derived MMPs contribute to cancer progression. Glioma M2-polarized macrophages induce glioma cell migration by the release of anti-inflammatory cytokines like TGF-β, the upregulation of MMP-2 and the suppression of TIMP-2 (Guadagno et al. 2018; Wick et al. 2001). Moreover, microglial TLR2 induces the upregulation of a membrane-anchored MMP (MT1-MMP) promoting cancer expansion and progression in an experimental mouse model of glioma (Vinnakota et al. 2013; Zhang et al. 2021). The expression of all four members of the TIMP family is upregulated in GBM, compared to the levels present in the normal brain and in IDH-mutant gliomas. High expression of both TIMP3 and TIMP4 can be a predictor of better survival in GBM patients. Interestingly, gene expression levels of TIMP family members positively correlate with immune cell infiltration in patients with GBM. TIMP1 expression correlates with dendritic cells, TIMP2 and TIMP3 expression is associated with B cells, CD4+T cells, macrophages, and neutrophils and TIMP4 expression is positively associated with the infiltration of B cells, CD8+T cells, and macrophages (Han et al. 2021).

## Circulating ECM Proteins as Biomarkers

In the last decade, the identification of biomarkers by liquid biopsy has attracted considerable attention. Liquid biopsy has been described as the gold standard mostly for the reduced invasiveness and high affordability compared to tissue biopsy. More interestingly, liquid biopsy provides real-time cancer status information giving the opportunity to monitor therapy response. Most of the research point to the investigation of three types of liquid biopsy markers, i.e., cell-free nucleic acids, extracellular vesicles (EVs), and circulating tumor cells (CTCs) (Irmer et al. 2023).

Recently, circulating ECM proteins have emerged as a promising biomarker of cancer progression. The fact that ECM components are actively released into blood during all the phases of tumorigenesis gives us the possibility to track lesions over time, from initiation to metastatic progression. Regarding the mechanisms by which ECM proteins or specific fragments migrate through the BBB to reach the circulation, BBB breakdown occurring in the glioma environment is sufficient to allow the dissemination of ECM components. However, according to Ghantasala et al (2020) this process is triggered only in high-grade gliomas, so most of the serum studies in gliomas have been carried out on serum/plasma specimens from GBM patients (Ghantasala et al. 2020). Interestingly, the increased vascular permeability induced by the cancer cells-released cytokines, chemokines, and microRNAs could also contribute (Zhou et al. 2014; Tominaga et al. 2015).

To date, a full knowledge of the ECM components that enter circulation in glioma is still missing. Ghorbani et al (2023) in their paper reported 12 plasma proteins which were increased or decreased in gliomas patients compared to meningiomas patients (GFAP, NEFL, EDDM3B, PROK1, MMP3, CTRL, GP2, SPINT3, FABP4, ALDH3A1, IL-12B, and OXT), however ECM proteins are not described. Similarly, none of the ECM proteins considered in this review resulted differently expressed in the serum of glioma patients in two major comprehensive reviews (Kalinina et al. 2011; Ghantasala et al. 2020).

Nevertheless, more recent investigations have described an altered serum level of Tenascin C, Collagen IV, COL1A2, and Laminin-5, as highlighted earlier. While the role of ECM proteins in the establishment of the general immune landscape of tumor microenvironment is widely accepted, recent observations suggest that their release in the serum of glioblastoma patients induces a systemic immunosuppression, also confirmed by the reduced effector activity of circulating T cells (Mirzaei et al. 2018). Overall, these considerations suggest that serum level of ECM proteins, alone or in combination, could represent a valuable non-invasive biomarker for glioma. Moreover, serum level of ECM proteins can also be an indicator of disease progress and drug therapeutic efficacy, and it can be useful in longitudinal studies.

## Discussion

In this review we shed light on recent advances regarding the role of ECM in modulating the crosstalk between neoplastic cells and immune cells in the context of AATs. ECM produced by distinct cell populations in AATs strongly dictate the behavior of immune cells.

Almost all types of immune cells can be found in AATs. In astrocytic tumors, they are present and influence many aspects of tumor biology above all via their interactions with the ECM. Recently, the specific immune cell type proportions as well as immune cell specific gene expression profiles have received considerable attention since the discovery of their potential to predict patient outcome and therapy responsiveness.

Cells of innate and adaptive immunity in AATs can destroy tumors, hold them to minimal progression or induce tumorigenic expansion. The specific composition of ECM and its structural organization can alter this balance (Table 3). However, most of the studies concerning the interactions between ECM and immunity cells in AATs concern macrophages and lymphocytes, probably due to their predominance in tumor environment. Accordingly, the action of such immune cells is responsible for the immunosuppressive “cold” microenvironment in GBM (Guadagno et al. 2018; Codrici et al. 2022).

**Table 3:** Immune cells in AATs

Immune cells	Tumorigenesis in AATs	Interaction with ECM in AATs
M1 macrophages	Against	Laminin activates microglia in proinflammatory phenotype
Cytotoxic T cells	Against	Hyaluronic acid determines increased infiltration Inhibition by TNC FNDC3B is positively correlated with immune infiltration in gliomas, especially in the cytotoxic T cells and antitumor associated immune cells TIMP-4 is positively associated with infiltration
NK-cells	Against	Unknown
Antigen-presenting cells	Against	TIMP1 expression is positively associated with dendritic cells, TIMP2 and TIMP3 expression is associated with B cells, CD4+T cells, macrophages, and neutrophils and TIMP4 expression is positively associated with the infiltration of B cells, CD8+T cells, and macrophages
M2 macrophages	Support	Infiltration and PDL-1 expression increased by Hyaluronic acid Collagen induces polarization to M2 macrophages Periostin stimulates macrophages recruitment and polarization to M2 phenotype TGF-β produced by M2 macrophages facilitates glioma cell migration via the upregulation of MMP-2 and suppression of tissue inhibitor of MMP-2 TIMP2, TIMP3, and TIMP4 expression is associated with macrophages
Tregs	Support	Unknown
T-helper 2 cells	Support	TIMP2 and TIMP3 expression is associated with CD4+T cells
Neutrophils	Different for the various subsets Role not clear. Infiltration increases with AATs grade	TIMP2 and TIMP3 expression is associated with neutrophils infiltration Probable ECM remodeling via secreted mediators
Myeloid-Derived Suppressor Cells (MDSCs)	Support	Unknown
Mast Cells	Against	Probable ECM remodeling via secreted mediators

Cells of the monocyte-macrophage lineage in normal and pathologic tissues are characterized by high heterogeneity and plasticity. In fact, mononuclear phagocytes can respond to environmental stimuli with the acquisition of distinct functional phenotypes. In response to signals, macrophages may undergo classical M1 activation (stimulated by TLR ligands and IFN-γ) or alternative M2 activation (stimulated by IL-4/IL-13). M1 macrophages, according to the typical representative model, produce the toxic nitric oxide (NO), whereas M2 macrophages produce the trophic polyamines (Mills et al. 2000; Perrotta et al. 2011a, b). The M1 phenotype shows the expression of high levels of proinflammatory cytokines, high production of reactive nitrogen and oxygen intermediates, promotion of Th1 response, and microbicidal and tumoricidal activity. In contrast, M2 macrophages are involved in tissue remodeling and tumor progression and have immunoregulatory functions. However, it is known that macrophage polarization is a continuum that allows the macrophage to rapidly transition from one state to another and change polarization state (Martinez et al. 2014; Sica et al. 2012).

Many factors regulate the infiltration and polarization of the macrophage population in AATs. In astrocytic tumors, tumor grade determines the level of hypoxia which in turn stimulates macrophages recruitment and polarization toward an M2 phenotype (Guadagno et al. 2018). 2-hydroxyglutarate (2HG) is the major oncometabolite produced from IDH-mutant astrocytoma and is responsible for defective collagen maturation. Its expression accounts for the inhibition of chromatin-modifying enzymes, namely 2-oxoglutarate-dependent dioxygenases, and interferes with hypoxia-inducible factor (HIF) transcriptome reprogramming and mammalian target of rapamycin (mTOR) pathway, thus dysregulating gene expression and further promoting carcinogenesis (Ježek 2020; Russo et al. 2014; Waitkus et al. 2016). As above reported, the complex interactions between macrophages and the different molecules of the ECM play a pivotal role in establishing the number and the state of activity of these cells in AATs (Amălinei et al. 2007; Chou et al. 2016; Han et al. 2021; Kiyokawa and Wakimoto 2019; Kiyokawa et al. 2021; Larsen et al. 2020; Ma et al. 2019; Rømer et al. 2021; Scali et al. 2016; Shi et al. 2015).

Lymphocytes have been defined as dynamic regulators of glioma pathobiology (Cordell et al. 2022). As a component of the adaptive immune system, can be considered as the counterpart of macrophages of innate host defense mechanisms in the regulation of AATs development. The killing of tumor cells by CD8+T lymphocytes is the main immunological mechanism of protection also in AATs. T lymphocytes perform a surveillance function by recognizing and killing potentially malignant cells expressing peptides presented in association with MHC class I molecules. Specific CD8+responses require cross-presentation by dendritic cells. Tumor cells or antigens are taken up by dendritic cells, processed and then presented to CD8+T lymphocytes allowing their maturation in cytotoxic T cells (CTL). As for CD4+lymphocytes, CD8+T lymphocytes may also exert antitumor responses by producing cytokines capable of inducing the differentiation of naïve CD8+lymphocytes into effector and memory CTLs. Furthermore, helper lymphocytes produce TNF and IFN-γ which increase the expression of MHC class I molecules and then stimulate CTL-mediated lysis. Interestingly, IFN-γ stimulates macrophage polarization into M2 macrophages.

Currently, immunotherapeutic tools employed against brain cancers are based on immune checkpoint inhibitors (ICIs) and vaccine-mediated immunization. ICIs consist of monoclonal antibodies that neutralize immunosuppressive signaling and enhance immune responses against tumor cells targeting costimulatory and inhibitory molecules, which can regulate the activation and effector functions of T lymphocytes. Under physiological conditions, those regulatory circuits are essential for self-tolerance, but in many cases, they may be a pathologic tool in malignancies. ICIs include anti-programmed cell death protein-1 (PD-1), anti-programmed cell death ligand-1 (PD-L1) or anti-cytotoxic T lymphocyte–associated protein 4 (CTLA-4) (Vismara et al. 2019).

As we reviewed in this paper, lymphocytes trafficking in AATs is strongly regulated by molecules of ECM such as HA (Kiyokawa and Wakimoto 2019; Kiyokawa et al. 2021), collagen (Rømer et al. 2021; Scali et al. 2016), TNC (Ma et al. 2019), and TIMPs (Han et al. 2021).

Other cell types such as neutrophils and mast cells have a function that is still poorly defined in different types of cancers (Hedrick and Malanchi 2022; Nechushtan 2010). Nevertheless, we cannot exclude that both tumor-associated neutrophils (TANs) and mast cells establish interactions with the ECM responsible for the modulation of immune response and tumor growth. TANs granules contain serine proteases, neutrophil elastase, MMP-9, and cathepsin G which induce cancer cells proliferation in AATs (Hurt et al. 2017; Lin et al. 2021). Main mast cells mediators have been described in the tumor microenvironment, including chymase, tryptase, VEGF, Histamine, TNF-α, MMP2, MMP-9, TIMPs, NGF, and sphingosine-1-phosphate (Donato et al. 2014; Komi et al. 2020). However, the main effects of mast cells in AATs are inhibition of proliferation, invasiveness, and stemness (Attarha et al. 2017).

A relationship between the composition of AATs ECM and immune infiltration seems to be clear. We have attempted to examine the interaction between single molecules of the ECM in AATs and immune cells. It is evident, however, that the overall effect on tumor growth will depend on multilateral interactions. Recently, a risk signature to predict glioma prognosis using ECM-related genes, including Growth Factors, was built. The mRNA expression data obtained from The Cancer Genome Atlas database and the Genotype-Tissue Expression database have been used to screen differentially expressed genes from ECM-related genes. A high-risk group and a low-risk group were identified. In turn, immune infiltration was evaluated in relation to the possible patterns of ECM. Cell type identification by estimating relative subsets of RNA transcripts indicates low levels of naive B cells, activated memory CD4 T cells, regulatory T cells, gamma delta CD8 T cells, naive CD4 T cells, resting memory CD4 T cells, M0 macrophages, M1 macrophages, resting mast cells, and neutrophils in the high-risk group (Liu and Li 2021).

## Conclusion

In conclusion, the interactions between the ECM and the cells of the immune system in AATs are very complex and bidirectional. Over the years, mutual influences between specific ECM molecule and single immune cell have been described, however ECM-immune cells interactions in GBM are still “clouded in mistery,” as recently stated by Collado et al (2024). Accordingly, within the brain tumor microenvironment, there is a large heterogeneity and so is the variety of ECM function.

Given the high potential of immunotherapy to significantly improve outcomes for patients with glioma, the current goal is the understanding of ECM-immune landscape in AATs that could provide useful insights in the identification of new biomarkers for prognostic evaluations and therapeutic decisions.

## Data Availability

Data sharing not applicable.
